# Advanced chemometric methods for simultaneous quantitation of caffeine, codeine, paracetamol, and p-aminophenol in their quaternary mixture

**DOI:** 10.1038/s41598-024-52450-4

**Published:** 2024-01-24

**Authors:** Khadiga M. Kelani, Reham A. Fekry, Yasmin M. Fayez, Said A. Hassan

**Affiliations:** 1 Analytical Chemistry Department, Faculty of Pharmacy, Cairo University, Kasr El-Aini Street, Cairo, 11562 Egypt; 2 Analytical Chemistry Department, Faculty of Pharmacy, Modern University for Technology and Information, El-Hadaba El-Wosta, Mokatam, 5th District, Cairo, Egypt

**Keywords:** Analytical chemistry, Cheminformatics

## Abstract

Two different multivariate techniques have been applied for the quantitative analysis of caffeine, codeine, paracetamol and p-aminophenol (PAP) in quaternary mixture, namely, Partial Least Squares (PLS-1) and Artificial Neural Networks (ANN). For suitable analysis, a calibration set of 25 mixtures with various ratios of the drugs and PAP impurity were established using a 4-factor 5-level experimental design. The most meaningful wavelengths for the chemometric models were chosen using Genetic Algorithm (GA) as a variable selection technique. By using an independent validation set, the validity of the proposed methods was evaluated. A comparative study was established between the three multivariate models (PLS-1, GA–PLS and GA–ANN). The comparison between the various models revealed that the GA–ANN model was superior at resolving the highly overlapped spectra of this quaternary combination. The drugs were successfully quantified in their pharmaceutical dosage form utilizing the GA–ANN models.

## Introduction

**Figure 1:**
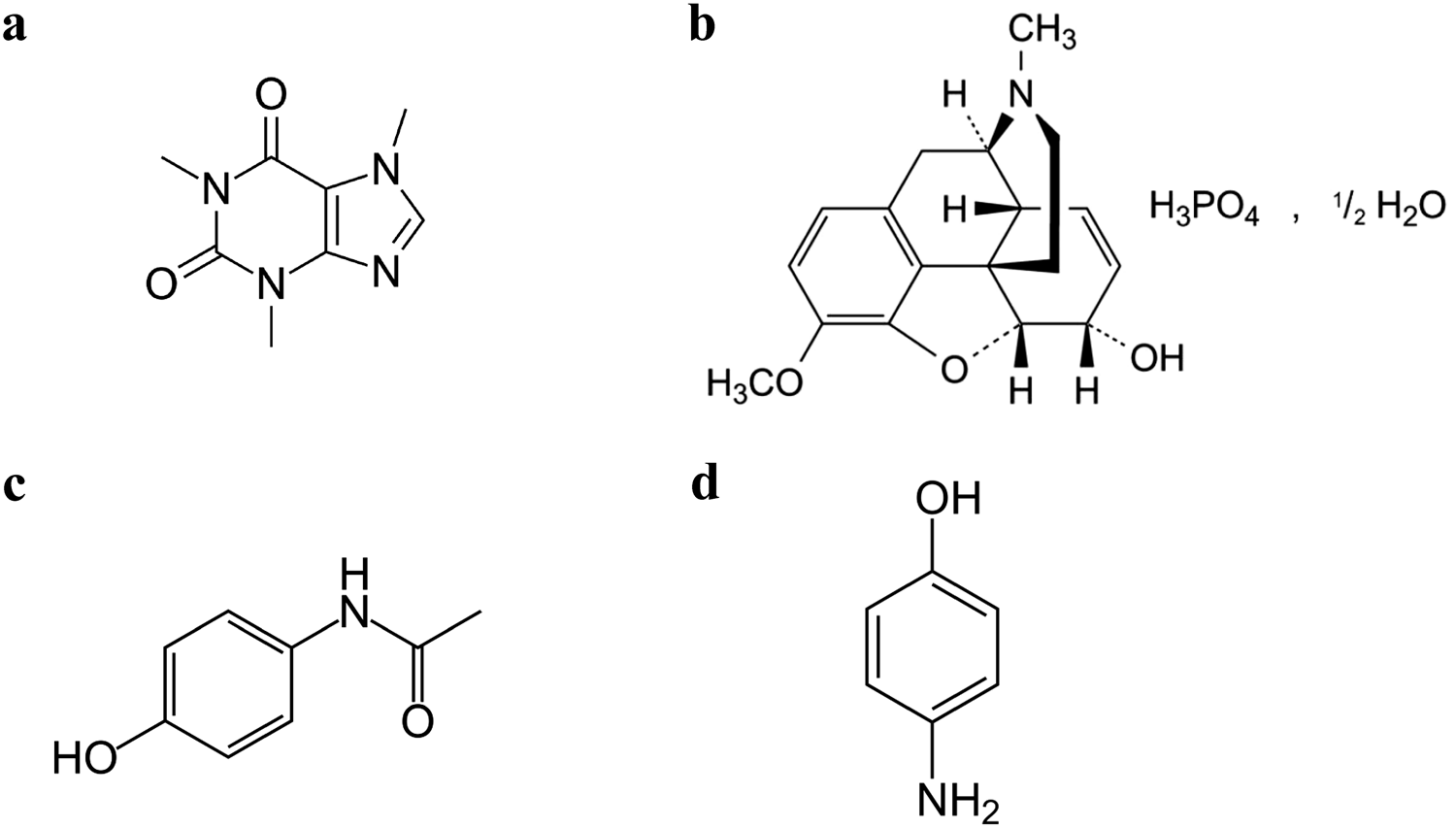
Structural formulae for (**a**) Caffeine (**b**) Codeine (**c**) Paracetamol and (**d**) p-aminophenol.

Caffeine (CAF) is 1,3,7-Trimethyl-3,7-dihydro-1*H*-purine-2,6-dione (Fig. 1a)^1^. It is a natural chemical with stimulant effects working by energising the heart, muscles and central nervous system as well as raising blood pressure. It can be found in approximately 60 different products, including coffee, tea, cola, cocoa, guarana and yerba mate^2^. Codeine phosphate hemihydrate (COD) is 4,5α-Epoxy-3-methoxy-17-methyl-7,8-didehydromorphinan-6α-ol phosphate hemihydrate (Fig. 1b)^3^. It is an opiate drug and considered as a prodrug of morphine used to treat pain, cough, and diarrhoea^4^. It is typically used to treat mild to moderate degrees of pain^5^. Paracetamol (PAR) is N-(4-hydroxyphenyl)acetamide (Fig. 1c)^1^, also known as acetaminophen. It is a medication that is used to alleviate pain and fever and commonly found in many cold medication^6^. It is present in many pharmaceutical dosage forms in mixtures with CAF, COD and other drugs. p-Aminophenol (PAP) (Fig. 1d) could be present in pharmaceutical dosage forms of PAR as degradation product or as a synthetic intermediate^7^, it is the official impurity K of PAR as stated by BP^1^. To ensure PAR safety, since PAP is reported to have severe teratogenic and nephrotoxicity effects^8^, a very low amount of PAP is allowed.

Several methods have been reported for analysis of CAF, COD, PAR and PAP in their different binary^9–12^ and ternary^13,14^ combinations or in their mixtures with other drugs^15–18^. In our previous work the quaternary mixture of CAF, COD, PAR and PAP was quantified simultaneously via validated HPLC and TLC methods^19^. There are no reported spectrophotometric or chemometric methods for determination of CAF, COD, PAR and PAP, simultaneously.

Currently, a key method for analysis of drug compounds is impurity testing. When submitting applications for new drug substances and new drug products, FDA recommends for adhering to the ICH guideline Q3A^20^. The Q3A guideline was updated and the most recent revision, Q3A(R2), added impurities in new compounds including inorganic and organic impurities as well as residual solvents^21^. Several analytical techniques have been widely used for pharmaceutical analysis and impurity testing such as chromatographic, spectrofluorimetric, and electrochemical methods^22–26^. Spectrophotometric techniques can be used as an alternative to chromatographic methods due to the prevalence of spectrophotometers, their ease of use and their lower cost. The main challenge is the spectral overlap between drugs and impurities, due to their structural similarity. Mathematical manipulations, derivative spectrophotometry and signal processing techniques offer the possibility of resolving the spectral overlap caused by this resemblance^27–31^.

Multivariate regression and design of experiment techniques have several applications in analytical chemistry^32–37^. Chemometrics are potent tools for processing spectral data and are anticipated to provide faster and more reliable results for resolving extremely overlapped spectra^38,39^. The determination of pharmaceuticals in the presence of their degradation products or impurities has been effectively accomplished using chemometric techniques^40–43^. Chemometric methods use diverse algorithms from multivariate regression such as Multivariate Curve Resolution- Alternating Least Squares (MCR-ALS) and Partial Least Squares (PLS) to machine learning techniques, e.g. artificial neural networks (ANN) and support vector machines (SVM). Details of how these algorithms work are out of the scope of this manuscript, however, they can be found in literature^44–47^. Variable selection is a common preprocessing technique used to improve the quality of data to enhance the prediction power of the chemometric algorithms^48^. Genetic algorithms (GA) apply survival of the fittest strategy among wavelengths to select the most significant ones for constructing the chemometric models^49^. It proved success in improving prediction power and reducing dimensionality of the data^50^.

The aim of work for this manuscript was the determination of CAF, COD, PAR and PAP simultaneously, in laboratory prepared mixtures and pharmaceutical dosage form using different chemometric methods. PLS and ANN were applied for analysis of the quaternary mixture to present a comparison between multivariate regressions and artificial intelligence algorithms, respectively. GA was applied before both PLS and ANN to present the effect of variable selection on chemometric models’ prediction.

## Material and methods

### Instrument and software

Double beam UV–vis spectrophotometer (Shimadzu, Kyoto, Japan) was used with UV 160 PC model and bundle software. Processing of absorption and derivative spectra was done using version 3.7 of the UV PC personal spectroscopy program (Shimadzu, Kyoto, Japan). Scans have been performed at intervals between 200.0 to 400.0 nm at 0.2 nm interval with 1.00 cm quartz cells.

The multivariate techniques were performed using MATLAB® 9.2.0.538062 (R2017a). PLS was performed using PLS toolbox 2.1 (Eigenvector Research Inc., Manson, USA), while GA and ANN were performed using MATLAB toolboxes.

### Chemicals and solvents

CAF, COD and PAR were obtained from RAMEDA co, Egypt, and their purities were measured and found to be 100.04%, 100.02% and 99.58%, respectively, according to BP and EP^1,3^. PAP was supplied from Adwic, Egypt. Its purity was examined and found to be 99.45% according to reported method^51^. Solpadeine® tablets were purchased from local market and labelled to contain 500, 30 and 8 mg of PAR, CAF and COD, respectively.

### Stock solutions

Standard solutions with concentration of 200 µg mL^−1^ of the cited compounds were prepared separately using distilled water.

### Procedures

#### Spectral characteristics of CAF, COD, PAR and PAP

The absorption spectra of 5 µg.mL^−1^ CAF, COD, PAR and PAP in water were recorded over the range of 200–400 nm, and water was utilised as a blank.

#### Construction of calibration and validation sets for the multivariate methods

The calibration set was constructed using five-level four-factor design^52^, where 25 mixtures containing various amounts of the cited medicines and PAP were included in the design. The design’s five levels were coded from −2 to 2 with centre levels of 3.6, 8, 12 and 4.5 µg.mL^−1^ for CAF, COD, PAR and PAP, respectively (Table 1). The designated quaternary mixtures were prepared by adding various aliquots of the previously prepared standard solutions to 10-mL flasks and completing the remaining volume with distilled water. Spectral data were collected in the range of 200–400 nm. 210–300 nm was the data range selected for CAF, COD and PAR, whereas 210–340 nm was selected for PAP, both with 1.0 nm intervals. The selected data was then sent to MATLAB for processing. Then, models for PLS-1, GA–PLS and GA–ANN were constructed. GA was used as a tool for variable selection to examine its impact on the models’ optimization. The prediction ability of the proposed models was tested and compared using an independent validation set of six mixtures that covered concentrations within the calibration ranges (Table 1).

**Table 1:** The concentration of COD, CAF, PAR and PAP in different mixtures used in the training and validation sets.

Mixture no	CAF (µg.mL^−1^)	COD (µg.mL^−1^)	PAR (µg.mL^−1^)	PAP (µg.mL^−1^)
1	3.6	8	12	4.5
2	3.6	2	4	7.5
3	1.2	2	20	3
4	1.2	14	8	7.5
5	6	5	20	4.5
6	2.4	14	12	3
7	6	8	8	3
8	3.6	5	8	6
9	2.4	5	16	7.5
10	2.4	11	20	6
11	4.8	14	16	4.5
12	6	11	12	7.5
13	4.8	8	20	7.5
14	3.6	14	20	1.5
15	6	14	4	6
16	6	2	16	1.5
17	1.2	11	4	4.5
18	4.8	2	12	6
19	1.2	8	16	6
20	3.6	11	16	3
21	4.8	11	8	1.5
22	4.8	5	4	3
23	2.4	2	8	4.5
24	1.2	5	12	1.5
25	2.4	8	4	1.5
26*	1.2	2	20	2
27*	3.6	8	12	4.5
28*	4	4	4	4
29*	6	2	16	1.5
30*	1.2	14	4	7.5
31*	6	14	4	1.5

*Mixtures of validation set.

#### Application of the proposed GA–ANN method for the simultaneous determination of CAF, COD and PAR in Solpadeine® tablets

Ten tablets of Solpadeine® were weighed and finely powdered. An accurately weighed portion of the powder equivalent to 500, 30 and 8 mg of PAR, CAF and COD, respectively, were put into a 100-mL beaker, sonicated in 30 mL distilled water for 10 min and filtered into a 100-mL volumetric flask. The residues were washed three times each using 10 mL of distilled water, and the solution was completed to the mark with the same solvent (Solution A). An aliquot of 0.4 mL was accurately transferred into a 100-mL volumetric flask, and the volume was completed with water (Solution B) to achieve final concentrations of 1.2, 0.32 and 20 µg.mL^−1^ for CAF, COD and PAR, respectively. COD in this solution was spiked to reach the linearity range of the methods. Aliquot equivalent to 200 µg COD was accurately transferred from its stock solution (200 µg mL^−1^) into a 100-mL volumetric flask to which 0.4 mL aliquot from Solution A was transferred to reach concentration of COD equals to 2.32 µg mL^−1^.

## Results and discussion

**Figure 2:**
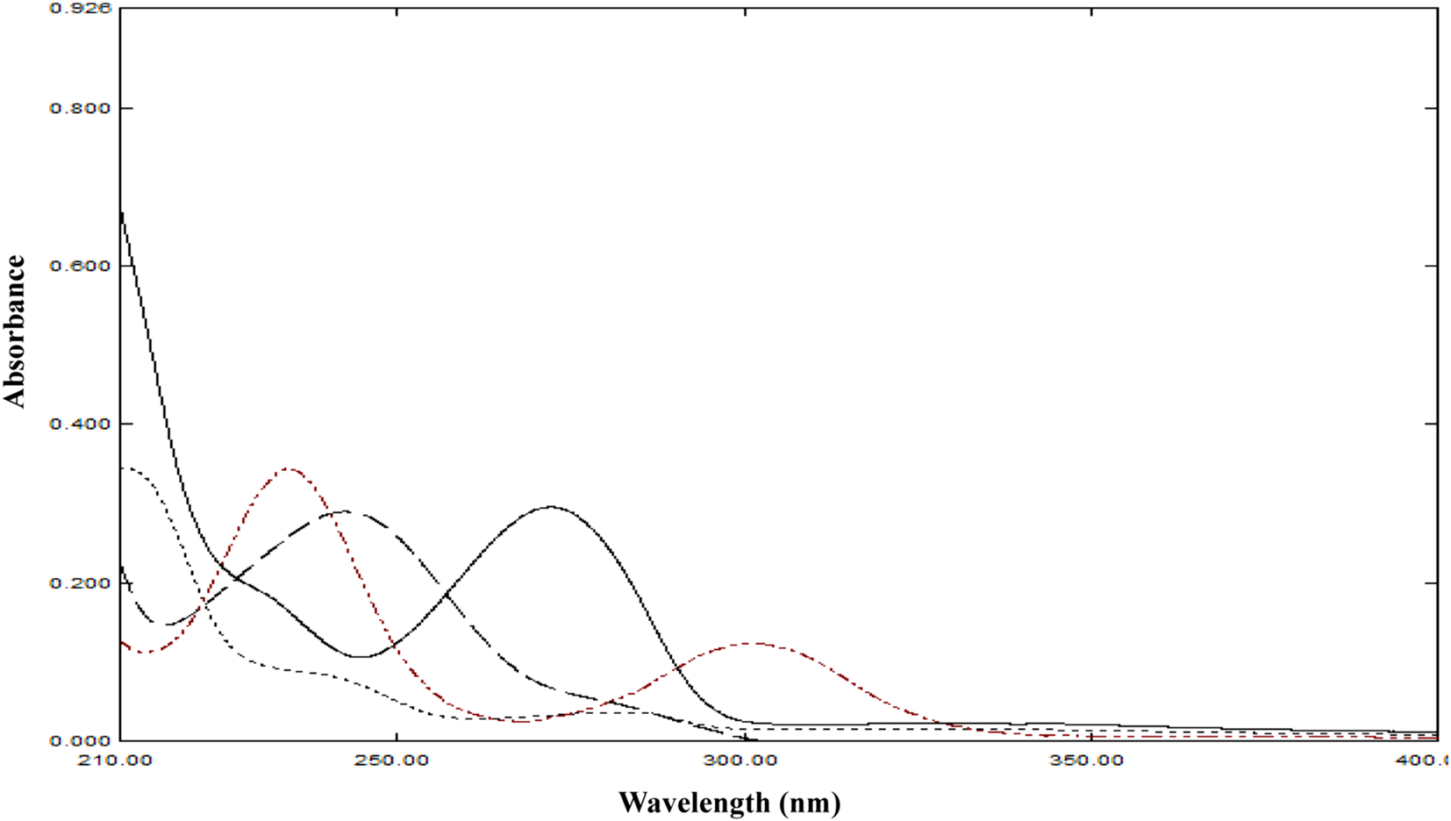
Zero order absorption spectra of 5 µg/mL CAF (─), COD (. . . .), PAR (-—-) and PAP (- . .—. . -) using methanol as blank.

In previous work, the quaternary mixture of CAF, COD, PAR and PAP was quantified using chromatographic methods^19^. According to literature, there are no reported spectrophotometric or chemometric methods for the simultaneous determination of this mixture. The spectra of CAF, COD and PAR along with PAP impurity show severe overlap as shown in Fig. 2. Spectral analysis becomes more challenging and the ability of traditional models to handle spectrophotometric data is lowered when the number of components in a mixture increases. That prevented traditional spectrophotometric methods from quantitation of this mixture, therefore the use of chemometrics to solve such spectral overlap was necessary.

### Calibration and validation sets

A calibration set was created using a five-level four-factor design with an independent validation set. The five concentration levels for each compound were chosen according to their spectral signals at the chosen wavelengths, so the absorbance of the mixtures of calibration, validation, and dosage form did not exceed the linearity of the spectrophotometer. Also, the ratio of the drugs in the dosage form was considered during the selection process. Therefore, the five levels were chosen in the concentration ranges of 1.2–6, 2–14, 4–20, and 1.5–7.5 μg mL^−1^ for CAF, COD, PAR, and PAP, respectively. The mixtures were measured, and their spectra were recorded between 200 and 400 nm. The designated data ranges are essential to reproduce accurate and precise models. Data points were chosen in the range of 210–300 nm for CAF, COD and PAR, while for PAP, the region selected was from 210–340 nm. The wavelengths below 210 nm were discarded due to high absorbance values that exceeded linearity and will represent noise to the models. The wavelengths longer than 300 and 340 nm were discarded as the corresponding drug’s spectrum shows no absorbance beyond these wavelengths (Fig. 2). The data was decomposed using principal component analysis (PCA) with the scores and loading plots are shown in Supp. Mat. (Fig. S1 and S2).

### Partial least squares-1

**Figure 3:**
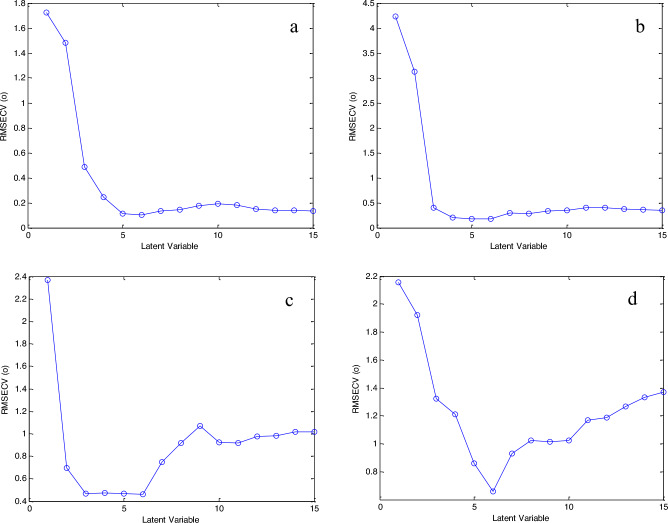
The optimum number of LVs of (**a**) CAF, (**b**) COD, (**c**) PAR and (**d**) PAP for PLS-1 models.

PLS-1 is a method for factor analysis. Being related to a single vector of dependent variables is its main difference from PLS-2. This improves the ability of prediction of complicated systems. An ideal number of latent variables (LV) for each single component is provided throughout the model development. This is preferable than using a single optimal number for all components together, which may be unreliable. The ideal number of LV must be taken into consideration to prevent the problem of overfitting^53^. The number of LV was chosen via leave one out cross validation method, and the best LVs were those of the least root mean square error of cross-validation (RMSECV). In this manuscript, 5, 4, 3 and 6 LVs were optimum for CAF, COD, PAR and PAP, respectively, as shown in Fig. 3. Before building the models, the data was either used as raw data or preprocessed using autoscaling or mean centering. Mean centering was the optimum preprocessing method, presenting better recoveries, RMSEP and RSD than other methods. The PLS-1 models could not expect the concentration of the four compounds in all validation set mixtures as shown in Table 2. This can be attributed to the severe overlap between the compounds, in addition to the contrast in their concentrations which are ranging from 1.2 to 20 μg mL^−1^. This is confirmed by the bad calibration data of the lines drawn between actual and predicted concentrations of validation set as shown in Table 3.

**Table 2:** Determination of COD, CAF, PAR and PAP in the validation set by the proposed models.

Mix. No	Concentration (µg.mL^−1^)				Recovery %^a,b^											
					PLS-1				GA–PLS				GA–ANN			
	CAF	COD	PAR	PAP	CAF	COD	PAR	PAP	CAF	COD	PAR	PAP	CAF	COD	PAR	PAP
26	1.2	2	20	2	99.62	98.8	97.74	**107.4**^**b**^	101.96	97.86	97.66	**105.07**^**b**^	102.03	98.14	98.64	102.25
27	3.6	8	12	4.5	98.59	101.07	98.5	102.73	97.56	99.65	97.66	100.91	97.67	97.96	98.83	102.02
28	4	4	4	4	**108.19**^**b**^	**105.83**^**b**^	**105.08**^**b**^	102.57	**105.93**^**b**^	**104.72**^**b**^	**105.38**^**b**^	100.67	102.15	101.77	102.56	101.8
29	6	2	16	1.5	100.99	**108.85**^**b**^	102.15	**95.19**^**b**^	101.78	**105.85**^**b**^	99.5	**95.81**^**b**^	102.44	102.05	99.82	98.07
30	1.2	14	4	7.5	**96.59**^**b**^	102.78	**104.66**^**b**^	102.81	**96.09**^**b**^	101.83	102.12	100.83	101.38	100.9	102.55	101.08
31	6	14	4	1.5	**96.71**^**b**^	99.83	102.53	**94.03**^**b**^	101.45	101.79	100.18	**96.06**^**b**^	101.58	101.45	102.57	98.53
	Mean				99.73	100.62	100.23	102.7	100.69	100.28	99.42	100.8	101.21	100.38	100.83	100.63
	SD				1.20	1.71	2.46	0.12	2.10	1.91	1.88	0.12	1.78	1.84	1.94	1.85
	RMSEP^c^				0.045	0.200	0.344	0.153	0.105	0.181	0.292	0.046	0.087	0.125	0.283	0.062

Significant values are in bold.

^a^Average of three determinations.

^b^Results were excluded according to rejection rule^55^.

^c^Root Mean Square Error of Prediction.

**Table 3:** Statistical parameter values for simultaneous determination of COD, CAF, PAR and PAP using the optimized chemometric methods.

Parameter of interest	PLS-1				GA–PLS				GA–ANN			
	CAF	COD	PAR	PAP	CAF	COD	PAR	PAP	CAF	COD	PAR	PAP
Concentration range (µg.mL^−1^)	1.2–6	2–14	4–20	1.5–7.5	1.2–6	2–14	4–20	1.5–7.5	1.2–6	2–14	4–20	1.5–7.5
No. of LV/hidden neurons	5	4	3	6	5	4	2	5	10	8	3	30
RMSEC^a^	0.068	0.125	0.219	0.129	0.044	0.091	0.238	0.109	0.045	0.091	0.186	0.084
RMSEP^b^	0.045	0.200	0.344	0.153	0.105	0.181	0.292	0.046	0.087	0.125	0.283	0.062
RMSECV^c^	0.081	0.134	0.269	0.159	0.058	0.095	0.270	0.173	–	–	–	–
Intercept^d^	0.0310	0.0977	0.2660	−0.0720	−0.0409	0.0149	0.2179	−0.0320	−0.0310	−0.0453	0.1798	−0.0222
Slope^d^	0.9958	1.0057	0.9768	1.0407	1.0257	1.0148	0.9695	1.0143	1.0214	1.0116	0.9798	1.0177
Correlation coefficient (r)^d^	0.9967	0.9996	0.9987	0.9995	0.9989	0.9998	0.9998	0.9997	0.9995	0.9998	0.9999	0.9999

^a^Root Mean Square Error of Calibration.

^b^Root Mean Square Error of Prediction.

^c^Root Mean Squares Error of Cross-Validation.

^d^Data of the straight line plotted between predicted concentrations of each component versus actual concentrations of validation set.

### Genetic algorithm optimization

**Figure 4:**
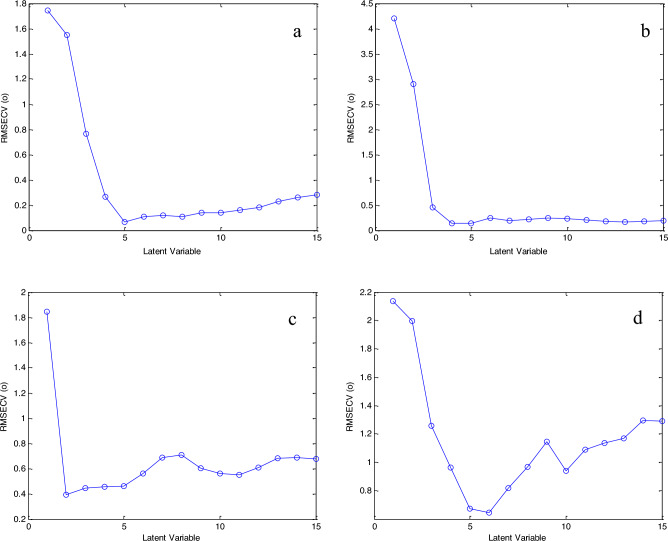
The optimum number of LVs of (**a**) CAF, (**b**) COD, (**c**) PAR and (**d**) PAP for GA–PLS models.

GA is an algorithm aroused by evolution and natural selection theory. The information is encoded using structures based on data that resemble chromosome. It aims to identify a starting population of solutions and then relies on survival of the fittest to allow the evolution of superior solutions^49^. It is mainly considered to be a function optimization technique. It enhances data selection to choose the most significant data points for getting the optimal outcome of the existing results. The GA’s set parameters are crucial for developing an effective selection model; therefore, several trials were conducted to optimize GA parameters. Different population sizes were tried (20, 50, 100, 150, and 200), and the best size regarding R% and RMSEP of the validation set was 100 for CAF and PAR, 200 for COD, and 20 for PAP. The number of variables per window varied from 2 to 20, and the optimum number of variables was 2 for all analytes. The number of LVs used in GA model construction was the optimum number obtained from PLS-1 models. In all fittings, the mutation rate was set to 0.005 using single breeding cross over and random cross-validation. Table 4 summarizes the optimized GA parameters. The data points number was dropped from 90 to 28, 30 and 34 for CAF, COD and PAR, respectively. Whereas the number was reduced from 130 to 44 for PAP. This means that, using GA, the data could be reduced to about 31–37% of the original data. These data points were used as inputs in PLS-1 and ANN because they are considered the most significant ones according to GA. The optimum number of LV for GA–PLS models are shown in Fig. 4. When GA was applied to PLS-1, the number of LVs didn’t differ for CAF and COD, but it was reduced for PAR and PAP, meaning GA improved the prediction power of these two models.

**Table 4:** Parameters of the genetic algorithms.

Parameter	Value
Population size	CAF, PAR (100) COD (200) PAP (20)
Maximum generations	50
Mutation rate	0.005
The number of variables in a window (window width)	2
Percent of population the same at Convergence	CAF, PAR, PAP (100) COD (50)
% wavelengths used at initiation	50
Crossover type	Single
Maximum number of latent variables	CAF (5) COD (4) PAR (3) PAP (6)
Cross validation	Random
Number of subsets to divide data into for cross validation	4
Number of iterations for cross validation at each generation	2

The GA–PLS models could not also expect the concentration of the four compounds in all validation set mixtures as shown in Table 2. Yet, it can be seen from the values of R%, SD, RMSEP and higher number of successful predictions in Table 2, that GA either enhanced predictive capabilities of PLS-1 models or provided equivalent predictive power. This suggests a better selectivity was attained by the GA models for these compounds and the calibration data in Table 3 confirmed this fact.

### Artificial neural networks

ANN works through a network of neuron-based structure, which consist of three layers: input, hidden and output. Here, feed-forward networks were used, and their learning process was accomplished by back propagation^46^. In order to avoid overfitting, it was discovered that one hidden layer was sufficient when building the neurons.

Through trial-and-error approach, several parameters were adjusted for the networks to get the highest possible prediction performance. These parameters include the number of neurons in the hidden layer, transfer function pairs and training functions.

The choice of the transfer function relies on the type of the analyzed data. In our work, different function pairs were examined purelin-purelin, tansig-purelin and logsig-purelin. By utilizing purelin-purelin function for all the analytes, the best results were obtained. This can be explained by the linear correlation between absorbance and concentration in the examined mixture. The networks were trained using a variety of training functions and with no variations in RMSEP between them, the Levenberg–Marquardt (TRAINLM) training function was selected to save time.

To prevent overfitting, the validation set was incorporated into the training process and the training was stopped when root mean square error (RMSE) of the calibration set decreased and that of validation set increased. Table 5 summarizes the optimal networks architectures.

**Table 5:** Optimized parameters of ANNs.

Method	GA–ANN			
Drug	CAF	COD	PAR	PAP
Hidden neurons number	10	8	3	30
Transfer functions	Purelin–Purelin			
Learning coefficient	0.001			
Learning coefficient decrease	0.001			
Learning coefficient increase	100			

**Figure 5:**
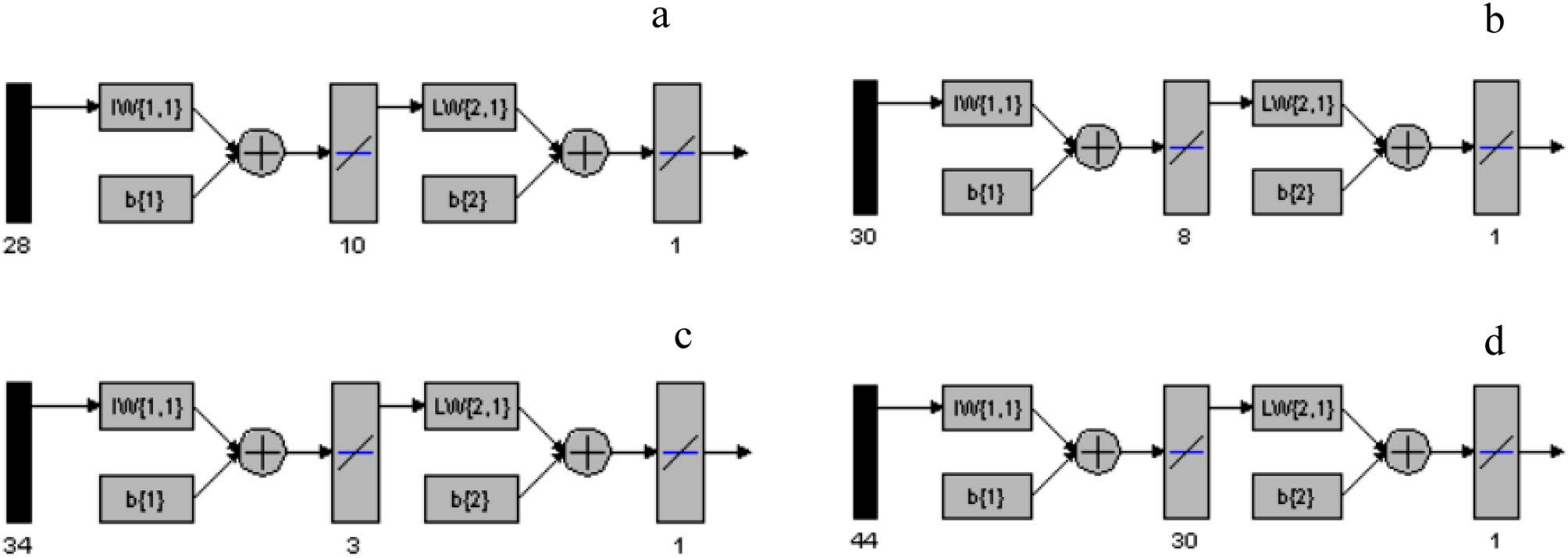
Different layers of the networks used for (**a**) CAF, (**b**) COD, (**c**) PAR and (**d**) PAP prediction using GA–ANN.

The neurons number in hidden layer was examined by training the networks and evaluating the resulting RMSEP. The neurons number for PAR in the hidden layer was only 3, while for PAP was 30 (Fig. 5). This can be attributed to the higher concentration of PAR in the mixtures in contrast to the low content of PAP impurity, which facilitated the prediction of PAR concentrations with a smaller number of neurons. Also, the fact that PAP model included higher number of variables (44) compared to the variables of PAR (34), which usually need more neurons for processing. This can be compared to previous results regarding the effect of number of variables on the number of hidden neurons in ANN models^54^.

Apparently, GA–ANN could expect the concentration of the four compounds successfully in all validation mixtures (Table 2). This can be attributed to the artificial intelligence nature of these models.

### Application of the proposed GA–ANN models on dosage form and statistical comparison

Showing the best prediction power among the three methods (Table 2), the proposed GA–ANN models were used to determine CAF, COD and PAR in Solpadeine® tablets and results are shown in Table 6. Statistics were used to compare the results obtained from application of the models on analysis of the compounds in pure powder with those produced by using the pharmacopeial methods^1,3^ for the analysis of COD, CAF and PAR and the reported method^51^ for the analysis of PAP in pure powder. As shown in Table 7, no significant difference was found, which confirm the predictive ability of GA–ANN models.

**Table 6:** Determination of CAF, COD and PAR in solpadeine® tablets by the proposed chemometric models.

Product	Drug	GA–ANN Method
		Recovery% ± SD^a^
Solpadeine® tablets	CAF	99.45 ± 1.19
	COD	99.19 ± 1.96
	PAR	99.89 ± 0.99

^a^Average of three determination.

**Table 7:** Statistical comparison for the results obtained by the proposed GA–ANN models and the pharmacopeial and reported methods for the analysis of COD, CAF, PAR and PAP in pure powder.

Parameter of interest	GA–ANN				Pharmacopeial method^1,3^^a^			Reported method^51^^b^
	CAF	COD	PAR	PAP	CAF	COD	PAR	PAP
Mean	100.04	100.02	99.58	99.54	100.24	100.12	99.88	99.67
SD	1.22	0.86	1.10	0.64	1.34	0.52	0.90	0.84
N	5	5	5	5	5	5	5	5
Variance	1.483	0.737	1.199	0.413	1.787	0.266	0.814	0.709
Student's *t* test^c^ (2.306)	0.253	0.229	0.476	0.273	–	–	–	–
F value^c^ (6.39)	1.205	2.769	1.474	1.716	–	–	–	–

^a^BP titrimetric methods for CAF and PAR, EP titrimetric method for COD.

^b^Colorimetric determination at 410 nm after reaction with 3-cyano-*N*-methoxypyridinium perchlorate.

^c^The values in the parenthesis are the corresponding theoretical values of t and F at *P* = 0.05.

## Conclusion

The complex mixture of COD, CAF, PAR and PAP was resolved by applying advanced chemometrics. GA has improved the prediction power of PLS-1 for the existing dataset, and when combined with ANN better results were obtained. The outcomes in this study support the use of the suggested method (GA–ANN) in the quality control analysis of COD, CAF and PAR combinations without the interference of PAP impurity. This raise hopes for applying chemometrics to analyse pure powder drugs and dosage forms whose spectra are highly overlapping, utilizing low-cost and simple equipment like spectrophotometers.

### Supplementary Information

Supplementary Information.

## Data Availability

The data analysed during the current study are available from the corresponding author on reasonable request.
