# Supplementary material for: Advanced chemometric methods for simultaneous quantitation of caffeine, codeine, paracetamol, and p-aminophenol in their quaternary mixture
Source: Sci Rep. 2024 Jan 24;14:2085. doi: 10.1038/s41598-024-52450-4 (PMC10808474; doi:10.1038/s41598-024-52450-4)
Supplement: Supplementary file 1 — Supplementary Information. [file 41598_2024_52450_MOESM1_ESM.pptx]

## Slide 1
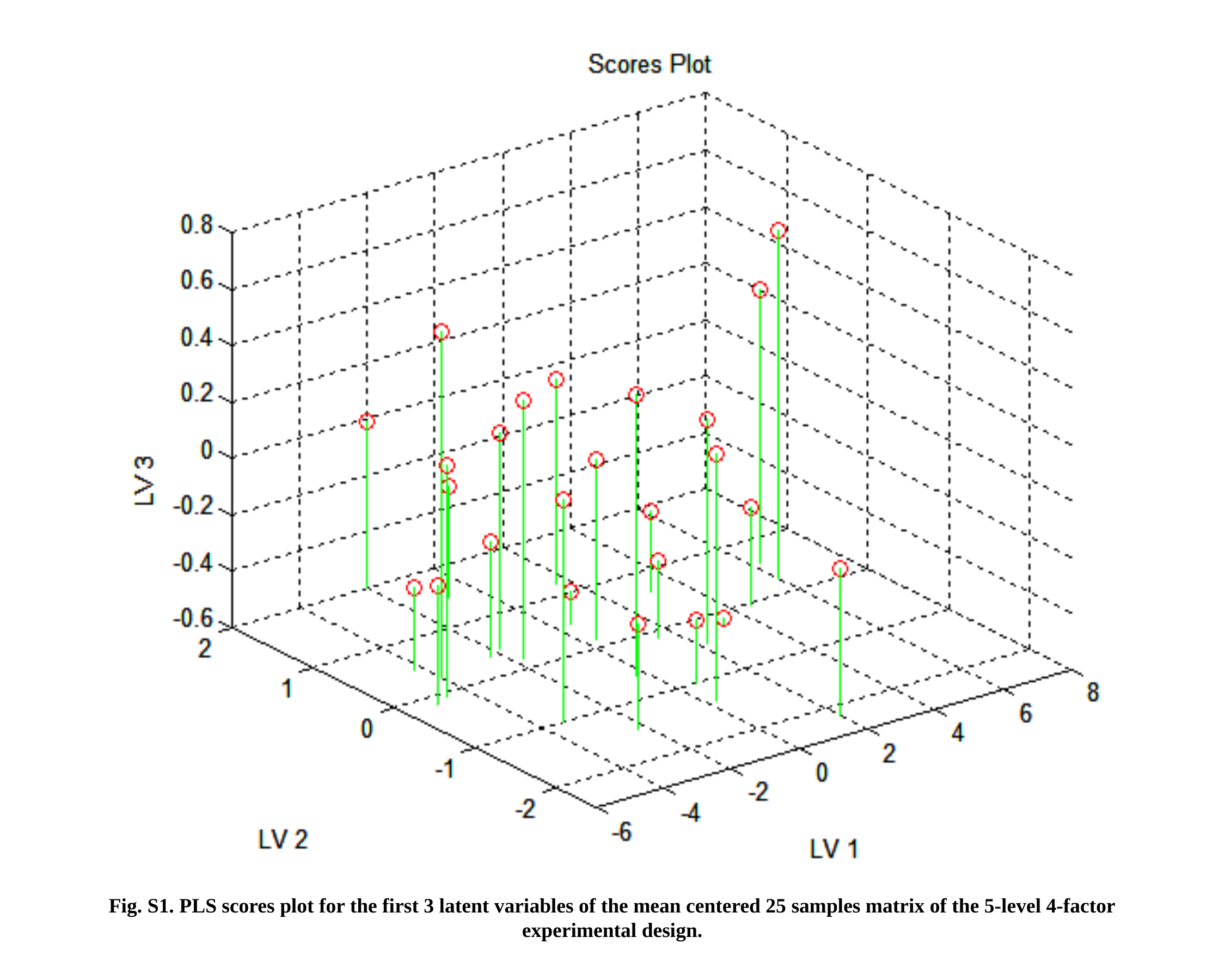

Fig. S1. PLS scores plot for the first 3 latent variables of the mean centered 25 samples matrix of the 5-level 4-factor experimental design.

## Slide 2
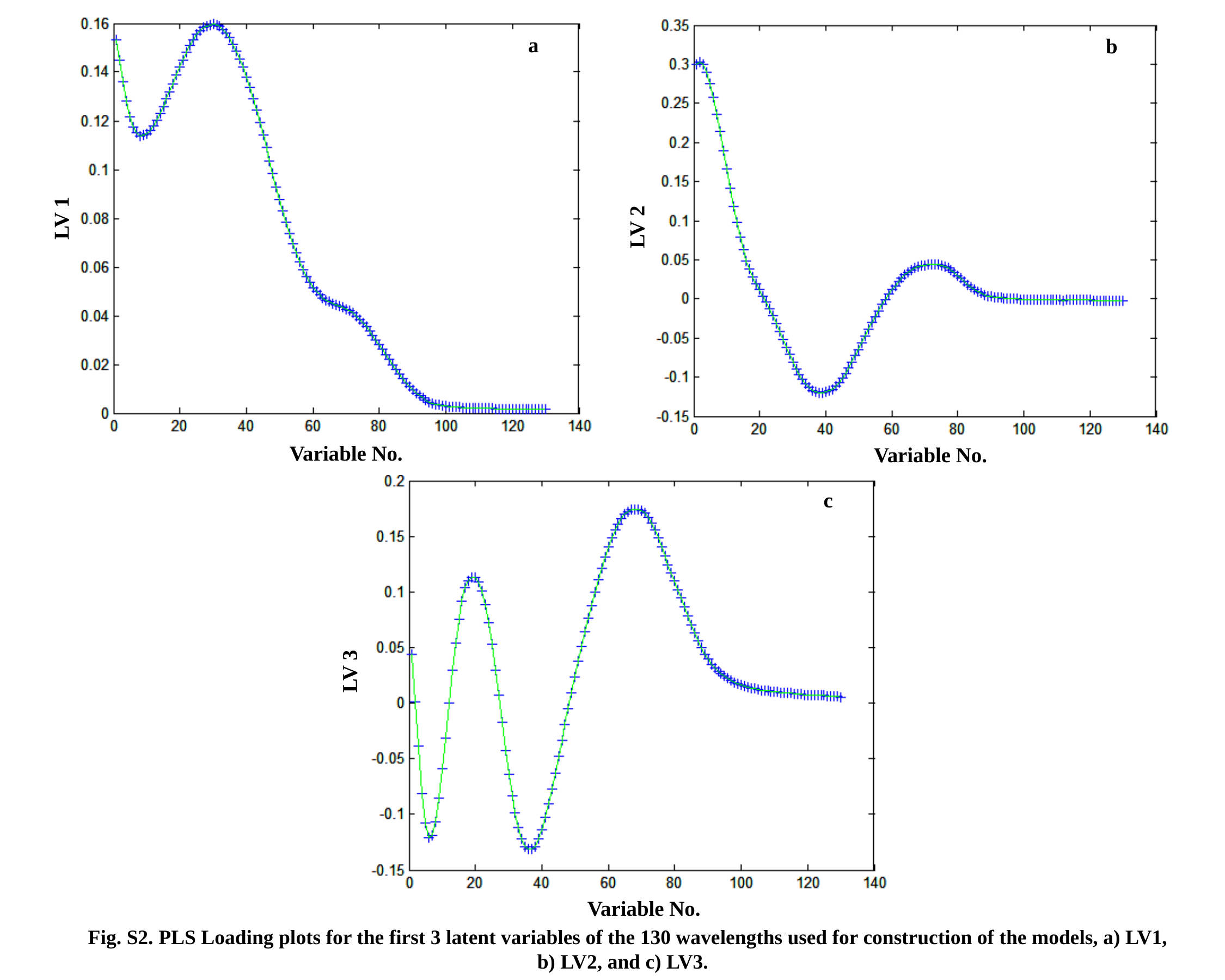

LV 1
Variable No.
a
LV 2
Variable No.
b
LV 3
Variable No.
c
Fig. S2. PLS Loading plots for the first 3 latent variables of the 130 wavelengths used for construction of the models, a) LV1, b) LV2, and c) LV3.
